# Supplementary material for: A new golden species of Diasporus (Anura: Eleutherodactylidae) from southwestern Colombia, with evaluation of the phylogenetic significance of morphological characters in Diasporus
Source: PeerJ. 2022 Feb 8;10:e12765. doi: 10.7717/peerj.12765 (PMC8833226; doi:10.7717/peerj.12765)
Supplement: Supplemental Information 1 [file peerj-10-12765-s001.docx]

>*Diasporus_lynchi_*sp_nov_CPZ_UV_7298_16S_GenBank_number_MZ871499

CTCCTGCTAAAACATAGTAGGTACAGCCTGCCCAGTGCCCAGTTTAACGGCCGCGGTATCCTAACCGTGCGAAGGTAGCGTAATCACCTGTTTTTTAATTTAAGACCTGTATGAATGGCATCACGAGAATCACACTGTCTCCTTTTTCTAATCAGTGAAACTAATCCCCCTGTGAAAAAGCAGGAATAATCTTACAAGACGAGAAGACCCTTTGGAGCTTTAGACAACGTGATGACCCCACAAACCACCTCATCCCAAGTCTTCGGTTGGGGCAACCACGGAGAAAAAAGAAACCTCCGAGACACACCTACAAAAGCCACACCTTTAAATATCAAAACTGACATTTATTGACCCAATATATGACCAACGAACCAAGTTACCCAAGGGATAACAGCGCAATCCACTTAGAGAGTCCCTATCGACAAGTGGGCTTACGACC

>*Diasporus_gularis_*CPZ_UV_7299_16S_GenBank_number_MZ871500

CAAAAACATCGCCTCCTGCTAAAATATAGTAGGTACAGCCTGCCCAGTGACCTATTTAACGGCCGCGGTATCCTAACCGTGCGAAGGTAGCGTAATCACCTGTTTTTTAATTAAAGACCTGTATGAATGGCATCACGAGAATCACACTGTCTCCTTTTTCTAATCAGTGAAACTAATCTCCCTGTGAAAAAGCAGGAATTAACCTACAAGACGAGAAGACCCTTTGGAGCTTTAGACAATGCGATGACCCCCTCCCACCACCTCGTCCCTAGTCTTCGGTTGGGGCAACCACGGAGAATAAAAGAACCTCCGAGATACAATCTACATAAAGCCACACCTTAAATATTTAAAACTAACATTAATTGACCCAGTATTCTGACCAACGAACCAAGTTACCCAAGGGATAACAGCGCAATCCACTTAGAGAGCCCCTATCGACAAGTGGGCTTACGACCTCGATGTTGGATCAAGATACCCAAGTGGTGCAGCCGCTACTAATGGTTCGTTTGTTCAACGATTAAAATCTCACGTGATC

>*Diasporus_gularis_*CPZ_UV_7299_COI_GenBank_number_MZ881958

GAGCAGCCCGGCTCATTACTGGGCGACGACCAAATCTACAATGTCCTTGTCACGGCCCACGCCTTTATCATGATTTTCTTTCTAGTCATGCCCGTAATGATCGGGGGCTTTGGTAACTGACTTGTCCCGTTAATACTGGGTGCACCCGATATGGCTTTCCCACGAATAAACAACATAAGCTTCTGGCTACTTCCCCCCTCTCTCCTTCTTCTCTTAGGCTCCGCCTGAGTAGAAGCTGGTGCTGGTACAGGTTGAACCGTATACCCCCCCTTAGCGAGCAACCTTGCTCATGCCGGCCCGTCAGTAGATATAACCATCTTCTCTCTGCACCTGGCAGGTGCTTCATCTATTATAGGAGCTGTAAACTTCATCACCACCATCTTTAACATAAAACCCCCCTCTTTCACCCAATACTTCACCCCCTTATTTGTCTGATCCGTCTTAATTACTGCAGCCCTCCTACTCCTATCACTTCCTGTCTTGGCAGCTGGGATCACTATACTTCTCACAGATCGTAACCTAAACACCACCTTCTTC
