# Supplementary material for: A new golden species of Diasporus (Anura: Eleutherodactylidae) from southwestern Colombia, with evaluation of the phylogenetic significance of morphological characters in Diasporus
Source: PeerJ. 2022 Feb 8;10:e12765. doi: 10.7717/peerj.12765 (PMC8833226; doi:10.7717/peerj.12765)
Supplement: Supplemental Information 2 — Species in bold are those for which we provide original sequences. COI, cytochrome oxidase subunit I; RAG1, recombinase activation. [file peerj-10-12765-s002.docx]

| Species | Voucher | 16S | COI | RAG1 |
| --- | --- | --- | --- | --- |
| *Adelophryne adiastola* | AJC 2463 | JX298299 | JX298340 | JX298167 |
| *Adelophryne baturitensis* | MTR 14013 | JX298281 | JX298321 | JX298149 |
| *Adelophryne gutturosa* | PK 2231 | JX298300 | JX298341 | JX298168 |
| *Adelophryne maranguapensis* | CFBHT 14119 | JX298286 | JX298326 | JX298153 |
| *Adelophryne pachydactyla* | MTR 16244 | JX298294 | JX298335 | JX298161 |
| *Adelophryne patamona* | PK 1969 | JX298296 | JX298337 | JX298163 |
| *Diasporus* aff *diastema* | SMF 97289 | – | KT186586 | KT119474 |
| *Diasporus* aff *diastema* | MHCH 2805 | – | KT186555 | KT119461 |
| *Diasporus* aff *diastema* | MHCH 2811 | – | KT186571 | KT119459 |
| *Diasporus* aff *diastema* | MHCH 2802 | KT186617 | KT186563 | KT119472 |
| *Diasporus* aff *diastema* | MHCH 2807 | – | KT186580 | KT119438 |
| *Diasporus* aff *diastema* | MHCH 2808 | KT186627 | KT186578 | KT119439 |
| *Diasporus* aff *diastema* | MHCH 2801 | KT186624 | KT186572 | KT119471 |
| *Diasporus* aff *hylaeformis* | UCR 16264 | JN991418 | JN991347 | – |
| *Diasporus* aff *hylaeformis* | MVZ203844 | EU186682 | – | EU186752 |
| *Diasporus* aff *hylaeformis* | MHCH 2859 | KT186614 | KT186558 | – |
| *Diasporus* aff *hylaeformis* | USNM 572456 | FJ784369 | FJ766810 | – |
| *Diasporus* aff *hylaeformis* | MVUP 1826 | FJ784390 | FJ766809 | – |
| *Diasporus* aff *hylaeformis* | SMF 89875 | JQ927340 | – | – |
| *Diasporus* aff *hylaeformis* | SMF 89872 | JQ927339 | – | – |
| *Diasporus* aff *hylaeformis* | SMF 89869 | JQ927338 | – | – |
| *Diasporus* aff *hylaeformis* | SMF 89868 | JQ927337 | – | – |
| *Diasporus* aff *quidditus* | SMF 97298 | KT186625 | – | KT119473 |
| *Diasporus* aff *quidditus* | MHCH 2825 | – | KT186560 | KT119454 |
| *Diasporus* aff *quidditus* | USNM 572444 | FJ784326 | – | – |
| *Diasporus* aff *quidditus* | MVUP 1832 | FJ784405 | – | – |
| *Diasporus* aff *quidditus* | SMF 97292 | KT186634 | KT186589 | KT119457 |
| *Diasporus* aff *quidditus* | SMF 97291 | KT186613 | – | KT119455 |
| *Diasporus* aff *quidditus* | MHCH 2824 | KT186621 | KT186569 | KT119443 |
| *Diasporus* aff *quidditus* | CH 6804 | KR863216 | KR862961 | – |
| *Diasporus* aff *quidditus* | AJC 1789 | KR863218 | KR862963 | – |
| *Diasporus* aff *quidditus* | CH 6648 | KR863212 | KR862957 | – |
| *Diasporus* aff *quidditus* | CH 6803 | KR863219 | KR862964 | – |
| *Diasporus amirae* | UCR 22254 | MK457182 | MK471388 | – |
| *Diasporus amirae* | UCR 21843 | MK457181 | MK471387 | – |
| *Diasporus amirae* | UCR 21842 | MK457180 | – | – |
| *Diasporus citrinobapheus* | SMF 89820 | JQ927334 | – | – |
| *Diasporus citrinobapheus* | SMF 89814 | JQ927333 | – | – |
| *Diasporus citrinobapheus* | MHCH 2371 | JQ927336 | – | – |
| *Diasporus citrinobapheus* | MHCH 2370 | JQ927335 | – | – |
| *Diasporus citrinobapheus* | USNM 572442 | FJ784425 | – | – |
| *Diasporus citrinobapheus* | USNM 572443 | FJ784484 | – | – |
| *Diasporus citrinobapheus* | MVUP 1830 | FJ784395 | – | – |
| *Diasporus citrinobapheus* | USNM 572455 | FJ784424 | – | – |
| *Diasporus citrinobapheus* | USNM 572454 | FJ784423 | – | – |
| *Diasporus citrinobapheus* | MVUP 1783 | FJ784338 | – | – |
| *Diasporus darienensis* | MHCH 2850 | KT186626 | KT186576 | KT119460 |
| *Diasporus darienensis* | MHCH 2841 | KT186618 | – | KT119449 |
| *Diasporus darienensis* | SMF 97662 | – | KT186564 | KT119447 |
| *Diasporus darienensis* | CH 6431 | KR863222 | KR862967 | – |
| *Diasporus darienensis* | CH 6425 | KR863223 | KR862968 | – |
| *Diasporus darienensis* | SMF 97305 | – | KT186582 | KT119444 |
| *Diasporus darienensis* | MHCH 2845 | – | KT186561 | KT119456 |
| *Diasporus darienensis* | SMF 97304 | – | KT186581 | KT119445 |
| *Diasporus darienensis* | MHCH 2862 | – | KT186579 | KT119458 |
| *Diasporus diastema* | CH 6676 | KR863214 | KR862959 | – |
| *Diasporus diastema* | SMF 97290 | – | KT186577 | KT119481 |
| *Diasporus diastema* | CH 6786 | KR863213 | KR862958 | – |
| *Diasporus diastema* | SMF 97287 | – | KT186566 | KT119470 |
| *Diasporus diastema* | CH 6792 | KR863217 | KR862962 | – |
| *Diasporus diastema* | CH 6802 | KR863215 | KR862960 | – |
| *Diasporus diastema* | CG 6800 | KR863211 | KR862956 | – |
| ***Diasporus gularis*** | **CPZUV 7299** | **MZ871500** | **MZ881958** | **–** |
| ***Diasporus lynchi* sp. nov.** | **CPZUV 7298** | **MZ871499** | **–** | **–** |
| *Diasporus majeensis* | SMF 97293 | – | KT186565 | KT119440 |
| *Diasporus majeensis* | MHCH 2839 | KT186629 | – | KT119442 |
| *Diasporus pequeno* | MHCH 2828 | – | KT186556 | KT119475 |
| *Diasporus pequeno* | MHCH 2830 | – | KT186559 | KT119478 |
| *Diasporus pequeno* | SMF 97335 | – | KT186583 | KT119476 |
| *Diasporus pequeno* | SMF 97663 | – | KT186575 | KT119477 |
| *Diasporus pequeno* | SMF 97337 | – | KT186570 | KT119479 |
| *Diasporus sapo* | SMF 97330 | KT186630 | KT186584 | KT119466 |
| *Diasporus sapo* | MHCH 2855 | KT186619 | KT186568 | KT119464 |
| *Diasporus sapo* | MHCH 2858 | KT186628 | – | KT119467 |
| *Diasporus sapo* | MHCH 2856 | KT186616 | – | KT119465 |
| *Diasporus* sp. 1 | MHCH 2874 | KT186622 | – | KT119469 |
| *Diasporus* sp. 2 | SMF 97339 | – | KT186573 | – |
| *Diasporus* sp | SMF 97652 | – | KT186562 | – |
| *Diasporus* sp | MHCH 1678 | – | KT186574 | – |
| *Diasporus tigrillo* | UCR 22367 | KT438505 | – | – |
| *Diasporus tigrillo* | UCR 22364 | KT438502 | – | – |
| *Diasporus tigrillo* | UCR 22366 | KT438504 | – | – |
| *Diasporus tigrillo* | UCR 22368 | KT438506 | – | – |
| *Diasporus tigrillo* | UCR 22365 | KT438503 | – | – |
| *Diasporus tinker* | CH 6439 | KR863220 | KR862965 | – |
| *Diasporus tinker* | AJC 1866 | KR863221 | KR862966 | – |
| *Diasporus tinker* | SMF 97320 | KT186632 | KT186587 | – |
| *Diasporus tinker* | MHCH 2871 | KT186620 | – | KT119451 |
| *Diasporus tinker* | SMF 97327 | KT186635 | – | KT119450 |
| *Diasporus tinker* | MHCH 2873 | KT186615 | – | KT119453 |
| *Diasporus tinker* | MHCH 2872 | KT186623 | – | KT119452 |
| *Diasporus vocator* | FMNH 257769 | JN991419 | JN991348 | – |
| *Diasporus vocator* | UCR 20133 | KT438508 | – | – |
| *Diasporus vocator* | UCR 21857 | KT438507 | – | – |
| *Eleutherodactylus alcoae* | USNM 564977 | EF493382 | – | EF493406 |
| *Eleutherodactylus caribe* | USNM 314179 | EF493385 | – | EF493411 |
| *Eleutherodactylus cavernicola* | USNM 266357 | EF493614 | – | – |
| *Eleutherodactylus chlorophenax* | USNM 257730 | EF493589 | – | – |
| *Eleutherodactylus cochranae* | USNM 326775 | EF493555 | – | – |
| *Eleutherodactylus counouspeus* | USNM 329989 | EF493719 | – | EU186760 |
| *Eleutherodactylus dimidiatus* | USNM 564986 | EF493640 | – | – |
| *Eleutherodactylus erythrochomus* | AMCC 118110 | EU186711 | – | – |
| *Eleutherodactylus fuscus* | USNM 266380 | EF493618 | – | – |
| *Eleutherodactylus glandulifer* | USNM 564988 | EF493655 | – | – |
| *Eleutherodactylus hypostenor* | USNM 257731 | EF493585 | – | – |
| *Eleutherodactylus inoptatus* | USNM 331931 | EF493380 | – | HQ831826 |
| *Eleutherodactylus interorbitalis* | CIG 584 | MG856995 | – | – |
| *Eleutherodactylus longipes* | CIG 611 | MG857006 | – | – |
| *Eleutherodactylus marnockii* | USNM 331345 | DQ283101 | – | – |
| *Eleutherodactylus martinicensis* | USNM 565001 | EF493343 | – | EF493419 |
| *Eleutherodactylus minutus* | USNM 331987 | EF493578 | – | – |
| *Eleutherodactylus nebulosus* | CIG 753 | MG857056 | – | – |
| *Eleutherodactylus nortoni* | USNM 257744 | EF493588 | – | – |
| *Eleutherodactylus ruthae* | USNM 257752 | EF493586 | – | – |
| *Eleutherodactylus saxatilis* | TJD 895 | MG857069 | – | – |
| *Eleutherodactylus verrucipes* | CIG 813 | MG857079 | – | – |
| *Ischnocnema lactae* | MTR 10435 | JX267308 | MN900830 | JX267627 |
| *Phyzelaphryne miriamae* | SMS 629 | JX298303 | JX298343 | JX298171 |
| *Phyzelaphryne nimio* | MCP 13687 | MG572227 | – | – |
