# Supplementary material for: A new golden species of Diasporus (Anura: Eleutherodactylidae) from southwestern Colombia, with evaluation of the phylogenetic significance of morphological characters in Diasporus
Source: PeerJ. 2022 Feb 8;10:e12765. doi: 10.7717/peerj.12765 (PMC8833226; doi:10.7717/peerj.12765)
Supplement: Supplemental Information 3 [file peerj-10-12765-s003.docx]

*Adelophryne adiastola.—*PERU: Loreto: Quebrada Vasquez, N side of lower Rio Tahuayo (KU 220564).

*Diasporus* aff. *diastema.—*COSTA RICA: Alajuela: Laguna Monte Alegre (KU 65742). Cartago: Tapanti (KU 65734).

*Diasporus antrax.—*COLOMBIA: Caldas: Municipio Norcasia (formerly part of Samaná), at campamento La Miel II, near junction of quebrada Tasajos with Río La Miel, Km 23 Carretera la Victoria – Samaná (ICN 41696).

*Diasporus gularis.—*COLOMBIA: Valle del Cauca: Municipio Buenaventura, Centro Forestal Bajo Calima (ICN 45169, 45171).

*Diasporus quidditus.—*COLOMBIA: Valle del Cauca: Municipio Buenaventura, Centro Forestal Bajo Calima (ICN 45173).

*Diasporus tinker.—*COLOMBIA: Valle del Cauca: Municipio Buenaventura, Centro Forestal Bajo Calima (ICN 45174, 45177, 45181).

*Diasporus vocator.—*COSTA RICA: Puntarenas: Agua Buena, Canton de Golfito (KU 37001).

*Eleutherodactylus alcoae.—*DOMINICAN REPUBLIC: Pedernales: 4 km NE Cabo Rojo (KU 265984).

*Eleutherodactylus counouspeus.—*HAITI: Grand'Anse: Castillon, S Marche Leon (KU 281372); Grotte La Foret, ca 9 km (airline) WSW Jeremie (KU 281373).

*Eleutherodactylus dimidiatus.—*JAMAICA: St. James: 0.8 mi W Mocho (KU 278708).

*Eleutherodactylus fuscus.—*JAMAICA: St. James: 0.8 mi W Mocho (KU 278708).

*Eleutherodactylus glandulifer*. HAITI: Grand'Anse: ca 2 km (airline) S Castillon (KU 278788).

*Eleutherodactylus hypostenor.—*DOMINICAN REPUBLIC: Barahona: 10.5 mi S Cabral (KU 280046).

*Eleutherodactylus inoptatus.—*DOMINICAN REPUBLIC: 12-14 km W La Cienegas (KU 325025).

*Eleutherodactylus longipes.—*MEXICO: Tamaulipas: Cueva de la Capilla, 13.5 km NW Gomez Farias (KU 147309).

*Eleutherodactylus nebulosus.—*MEXICO: Chiapas: 1.3 km N Puerto Madero (KU 58911).

*Eleutherodactylus nortoni.—*DOMINICAN REPUBLIC: Pedernales: 7 km N Cabeza de Agua (KU 283444).

*Eleutherodactylus ruthae.—*HAITI: Grand'Anse: Caye Morbette, 3.2 mi SW Paillant (KU 285925).

*Eleutherodactylus saxatilis.—*MEXICO: Sinaloa: 8 mi W El Palmito (KU 63330).

*Eleutherodactylus verrucipes.—*MEXICO: Hidalgo: 6 mi NE Jacala, Puerto de la Zorra (KU 60241).
