# Supplementary material for: A new golden species of Diasporus (Anura: Eleutherodactylidae) from southwestern Colombia, with evaluation of the phylogenetic significance of morphological characters in Diasporus
Source: PeerJ. 2022 Feb 8;10:e12765. doi: 10.7717/peerj.12765 (PMC8833226; doi:10.7717/peerj.12765)
Supplement: Supplemental Information 4 [file peerj-10-12765-s004.docx]

| Species | 0 | 1 |  | 2 | Source |
| --- | --- | --- | --- | --- | --- |
|  | Palmar tubercle shape | External vocal sacs |  | Coloration in life |  |
| *Adelophryne adiastola* AJC2463 | 0 | 1 |  | 0 | Hoogmoed and Lescure, 1984; Ortega-Andrade, 2009 |
| *Adelophryne baturitensis* MTR14013 | 0 | 1 |  | 0 | Hoogmoed *et al*., 1994 |
| *Adelophryne gutturosa* PK2231 | 0 | 1 |  | 0 | Hoogmoed and Lescure, 1984; MacCulloch *et al*. 2008 |
| *Adelophryne maranguapensis* CFBHT14119 | 0 | ? |  | 0 | Hoogmoed *et al*., 1994 |
| *Adelophryne pachydactyla* MTR16244 | 0 | 1 |  | 0 | Hoogmoed *et al*., 1994 |
| *Adelophryne patamona* PK1969 | 0 | ? |  | 0 | MacCulloch *et al*., 2008 |
| *Diasporus* 2 SMF97339 | ? | ? |  | ? |  |
| *Diasporus* aff *diastema* EPL MHCH2802 | 0 | 1 |  | 1 | Lynch, 2001; Batista *et al*., 2006 |
| *Diasporus* aff *diastema* EPL MHCH2805 | 0 | 1 |  | 1 | Lynch, 2001; Batista *et al*., 2006 |
| *Diasporus* aff *diastema* EPL MHCH2811 | 0 | 1 |  | 1 | Lynch, 2001; Batista *et al*., 2006 |
| *Diasporus* aff *diastema* EPL SMF97289 | 0 | 1 |  | 1 | Lynch, 2001; Batista *et al*., 2006 |
| *Diasporus* aff *diastema* MM MHCH2801 | 0 | 1 |  | 1 | Lynch, 2001; Batista *et al*., 2006 |
| *Diasporus* aff *diastema* MM MHCH2807 | 0 | 1 |  | 1 | Lynch, 2001; Batista *et al*., 2006 |
| *Diasporus* aff *diastema* MM MHCH2808 | 0 | 1 |  | 1 | Lynch, 2001; Batista *et al*., 2006 |
| *Diasporus* aff *hylaeformis* MHCH2859 | 0 | 1 |  | 0 | Lynch, 2001; Batista *et al*., 2006 |
| *Diasporus* aff *hylaeformis* MVUP1826 | 0 | 1 |  | 0 | Lynch, 2001; Batista *et al*., 2006 |
| *Diasporus* aff *hylaeformis* MVZ203844 | 0 | 1 |  | 0 | Lynch, 2001; Batista *et al*., 2006 |
| *Diasporus* aff *hylaeformis* SMF89868 | 0 | 1 |  | 0 | Lynch, 2001; Batista *et al*., 2006 |
| *Diasporus* aff *hylaeformis* SMF89869 | 0 | 1 |  | 0 | Lynch, 2001; Batista *et al*., 2006 |
| *Diasporus* aff *hylaeformis* SMF89872 | 0 | 1 |  | 0 | Lynch, 2001; Batista *et al*., 2006 |
| *Diasporus* aff *hylaeformis* SMF89875 | 0 | 1 |  | 0 | Lynch, 2001; Batista *et al*., 2006 |
| *Diasporus* aff *hylaeformis* UCR16264 | 0 | 1 |  | 0 | Lynch, 2001; Batista *et al*., 2006 |
| *Diasporus* aff *hylaeformis* USNM572456 | 0 | 1 |  | 0 | Lynch, 2001; Batista *et al*., 2006 |
| *Diasporus* aff *quidditus* AJC1789 | 0 | 1 |  | 0 | Lynch, 2001; Batista *et al*., 2006 |
| *Diasporus* aff *quidditus* CH6803 | 0 | 1 |  | 0 | Lynch, 2001; Batista *et al*., 2006 |
| *Diasporus* aff *quidditus* CH6804 | 0 | 1 |  | 0 | Lynch, 2001; Batista *et al*., 2006 |
| *Diasporus* aff *quidditus* MHCH_2825 | 0 | 1 |  | 0 | Lynch, 2001; Batista *et al*., 2006 |
| *Diasporus* aff *quidditus*MHCH2824 | 0 | 1 |  | 0 | Lynch, 2001; Batista *et al*., 2006 |
| *Diasporus* aff *quidditus* MVUP1832 | 0 | 1 |  | 0 | Lynch, 2001; Batista *et al*., 2006 |
| *Diasporus* aff *quidditus* SMF97291 | 0 | 1 |  | 0 | Lynch, 2001; Batista *et al*., 2006 |
| *Diasporus* aff *quidditus* SMF97292 | 0 | 1 |  | 0 | Lynch, 2001; Batista *et al*., 2006 |
| *Diasporus* aff *quidditus* SMF97298 | 0 | 1 |  | 0 | Lynch, 2001; Batista *et al*., 2006 |
| *Diasporus* aff *quidditus* USNM572444 | 0 | 1 |  | 0 | Lynch, 2001; Batista *et al*., 2006 |
| *Diasporus* aff *quidditus* CH6648 | 0 | 1 |  | 0 | Lynch, 2001; Batista *et al*., 2006 |
| *Diasporus amirae* UCR21842 | 0 | 1 |  | 0 | Arias *et al*., 2019 |
| *Diasporus amirae* UCR21843 | 0 | 1 |  | 0 | Arias *et al*., 2019 |
| *Diasporus amirae* UCR22254 | 0 | 1 |  | 0 | Arias *et al*., 2019 |
| *Diasporus citrinobapheus* MHCH2370 | ? | 1 |  | 1 | Hertz *et al*., 2012 |
| Dias*porus citrinobapheus* MHCH2371 | ? | 1 |  | 1 | Hertz *et al*., 2012 |
| *Diasporus citrinobapheus* MVUP1783 | ? | 1 |  | 1 | Hertz *et al*., 2012 |
| *Diasporus citrinobapheus* MVUP1830 | ? | 1 |  | 1 | Hertz *et al*., 2012 |
| *Diasporus citrinobapheus* SMF89814 | ? | 1 |  | 1 | Hertz *et al*., 2012 |
| *Diasporus citrinobapheus* SMF89820 | ? | 1 |  | 1 | Hertz *et al*., 2012 |
| *Diasporus citrinobapheus* USNM572442 | ? | 1 |  | 1 | Hertz *et al*., 2012 |
| *Diasporus citrinobapheus* USNM572443 | ? | 1 |  | 1 | Hertz *et al*., 2012 |
| *Diasporus citrinobapheus* USNM572454 | ? | 1 |  | 1 | Hertz *et al*., 2012 |
| *Diasporus citrinobapheus* USNM572455 | ? | 1 |  | 1 | Hertz *et al*., 2012 |
| *Diasporus darienensis* MHCH2845 | 0 | 1 |  | 0 | Batista *et al*., 2016 |
| *Diasporus darienensis* CH6425 | 0 | 1 |  | 0 | Batista *et al*., 2016 |
| *Diasporus darienensis* CH6431 | 0 | 1 |  | 0 | Batista *et al*., 2016 |
| *Diasporus darienensis* MHCH2841 | 0 | 1 |  | 0 | Batista *et al*., 2016 |
| *Diasporus darienensis* MHCH2850 | 0 | 1 |  | 0 | Batista *et al*., 2016 |
| *Diasporus darienensis* MHCH2862 | 0 | 1 |  | 0 | Batista *et al*., 2016 |
| *Diasporus darienensis* SMF97304 | 0 | 1 |  | 0 | Batista *et al*., 2016 |
| *Diasporus darienensis* SMF97305 | 0 | 1 |  | 0 | Batista *et al*., 2016 |
| *Diasporus darienensis* SMF97662 | 0 | 1 |  | 0 | Batista *et al*., 2016 |
| *Diasporus diastema* CH6676 | 0 | 1 |  | 0 | Lynch, 2001; Cope 1875 |
| *Diasporus diastema* CH6786 | 0 | 1 |  | 0 | Lynch, 2001; Cope 1875 |
| *Diasporus diastema* CH6792 | 0 | 1 |  | 0 | Lynch, 2001; Cope 1875 |
| *Diasporus diastema* CH6800 | 0 | 1 |  | 0 | Lynch, 2001; Cope 1875 |
| *Diasporus diastema* CH6802 | 0 | 1 |  | 0 | Lynch, 2001; Cope 1875 |
| *Diasporus diastema* SMF97287 | 0 | 1 |  | 0 | Lynch, 2001; Cope 1875 |
| *Diasporus diastema* SMF97290 | 0 | 1 |  | 0 | Lynch, 2001; Cope 1875 |
| *Diasporus gularis* CPZUV 7299 | 0 | 1 |  | 1 | Lynch, 2001; This study |
| *Diasporus lynchi* sp. nov. CPZUV 7298 | 0 | 1 |  | 1 | This study |
| *Diasporus majeensis* MHCH2839 | 0 | 1 |  | 0 | Batista *et al*., 2016 |
| *Diasporus majenesis* SMF97293 | 0 | 1 |  | 0 | Batista *et al*., 2016 |
| *Diasporus pequeno* MHCH2828 | 0 | 1 |  | 0 | Batista *et al*., 2016 |
| *Diasporus pequeno* MHCH2830 | 0 | 1 |  | 0 | Batista *et al*., 2016 |
| *Diasporus pequeno* SMF97335 | 0 | 1 |  | 0 | Batista *et al*., 2016 |
| *Diasporus pequeno* SMF97337 | 0 | 1 |  | 0 | Batista *et al*., 2016 |
| *Diasporus pequeno* SMF97663 | 0 | 1 |  | 0 | Batista *et al*., 2016 |
| *Diasporus sapo* MHCH2855 | 0 | – |  | 0 | Batista *et al*., 2016 |
| *Diasporus sapo* MHCH2856 | 0 | – |  | 0 | Batista *et al*., 2016 |
| *Diasporus sapo* MHCH2858 | 0 | – |  | 0 | Batista *et al*., 2016 |
| *Diasporus sapo* SMF97330 | 0 | – |  | 0 | Batista *et al*., 2016 |
| *Diasporus* sp 1 MHCH2874 | ? | ? |  | ? |  |
| *Diasporus tigrillo* UCR22364 | 0 | 1 |  | 1 | Savage, 1997; Lynch, 2001 |
| *Diasporus tigrillo* UCR22365 | 0 | 1 |  | 1 | Savage, 1997; Lynch, 2001 |
| *Diasporus tigrillo* UCR22366 | 0 | 1 |  | 1 | Savage, 1997; Lynch, 2001 |
| *Diasporus tigrillo* UCR22367 | 0 | 1 |  | 1 | Savage, 1997; Lynch, 2001 |
| *Diasporus tigrillo* UCR22368 | 0 | 1 |  | 1 | Savage, 1997; Lynch, 2001 |
| *Diasporus tinker* AJC1866 | 0 | 1 |  | 0 | Lynch, 2001 |
| *Diasporus tinker* CH6439 | 0 | 1 |  | 0 | Lynch, 2001 |
| *Diasporus tinker* MHCH2871 | 0 | 1 |  | 0 | Lynch, 2001 |
| *Diasporus tinker* MHCH2872 | 0 | 1 |  | 0 | Lynch, 2001 |
| *Diasporus tinker* MHCH2873 | 0 | 1 |  | 0 | Lynch, 2001 |
| *Diasporus tinker* SMF97320 | 0 | 1 |  | 0 | Lynch, 2001 |
| *Diasporus tinker* SMF97327 | 0 | 1 |  | 0 | Lynch, 2001 |
| *Diasporus vocator* FMNH257769 | 0 | 1 |  | 0 | Lynch, 2001; Batista *et al*., 2006 |
| *Diasporus vocator* UCR20133 | 0 | 1 |  | 0 | Lynch, 2001; Batista *et al*., 2006 |
| *Diasporus vocator* UCR21857 | 0 | 1 |  | 0 | Lynch, 2001; Batista *et al*., 2006 |
| *Diasporus vocator* MHCH1678 | 0 | 1 |  | 0 | Lynch, 2001; Batista *et al*., 2006 |
| *Diasporus* *vocator* SMF97652 | 0 | 1 |  | 0 | Lynch, 2001; Batista *et al*., 2006 |
| *Eleutherodactylus alcoae* USNM564977 | 0 | ? |  | ? | This study |
| *Eleutherodactylus caribe* USNM314179 | 0 | 1 |  | 0 | Hedges and Thomas, 1992; This study |
| *Eleutherodactylus cavernicola* USNM266357 | 0 | ? |  | 0 | Lynn, 1954 |
| *Eleutherodactylus chlorophenax* USNM257730 | ? | ? |  | ? |  |
| *Eleutherodactylus cochranae* USNM326775 | ? | ? |  | ? |  |
| *Eleutherodactylus counouspeus* USNM329989 | 0 | ? |  | ? | This study |
| *Eleutherodactylus dimidiatus* USNM564986 | 0 | ? |  | ? | This study |
| *Eleutherodactylus erythrochomus* AMCC118110 | 1 | ? |  | 0 | Palacios-Aguila and Santos-Bibiano, 2020 |
| *Eleutherodactylus fuscus* USNM266380 | 0 | ? |  | 0 | Lynn and Dent, 1943; This study |
| *Eleutherodactylus glandulifer* USNM564988 | 0 | – |  | ? | This study |
| *Eleutherodactylus hypostenor* USNM257731 | 0 | ? |  | ? | This study |
| *Eleutherodactylus inoptatus* USNM331931 | 0 | ? |  | ? | This study |
| *Eleutherodactylus interorbitalis* CIG584 | ? | ? |  | ? |  |
| *Eleutherodactylus longipes* CIG611 | 0 | ? |  | ? | This study |
| *Eleutherodactylus marnocki* USNM331345 | ? | ? |  | ? |  |
| *Eleutherodactylus martinicensis* USNM565001 | ? | ? |  | ? |  |
| *Eleutherodactylus minutus* USNM331987 | ? | ? |  | ? |  |
| *Eleutherodactylus nebulosus* CIG753 | 0 | 1 |  | ? | This study |
| *Eleutherodactylus nortoni* USNM257744 | 0 | – |  | ? | This study |
| *Eleutherodactylus ruthae* USNM257752 | 0 | ? |  | ? | This study |
| *Eleutherodactylus saxatilis* TJD895 | 0 | ? |  | ? | This study |
| *Eleutherodactylus verrucipes* CIG813 | 0 | ? |  | ? | This study |
| *Ischnocnema lactea* MTR10435 | 1 | – |  | 0 | Silva-Soares *et al*., 2018 |
| *Phyzelaphryne miriamae* SMS629 | 0 | 1 |  | 0 | Heyer, 1977; Hoogmoed and Lescure, 1984; Simões *et al*., 2018 |
| *Phyzelaphryne nimio* MCP13687 | 0 | 1 |  | 0 | Simões *et al*., 2018 |
