# Supplementary figures and images for: A new golden species of Diasporus (Anura: Eleutherodactylidae) from southwestern Colombia, with evaluation of the phylogenetic significance of morphological characters in Diasporus
Source: PeerJ. 2022 Feb 8;10:e12765. doi: 10.7717/peerj.12765 (PMC8833226; doi:10.7717/peerj.12765)

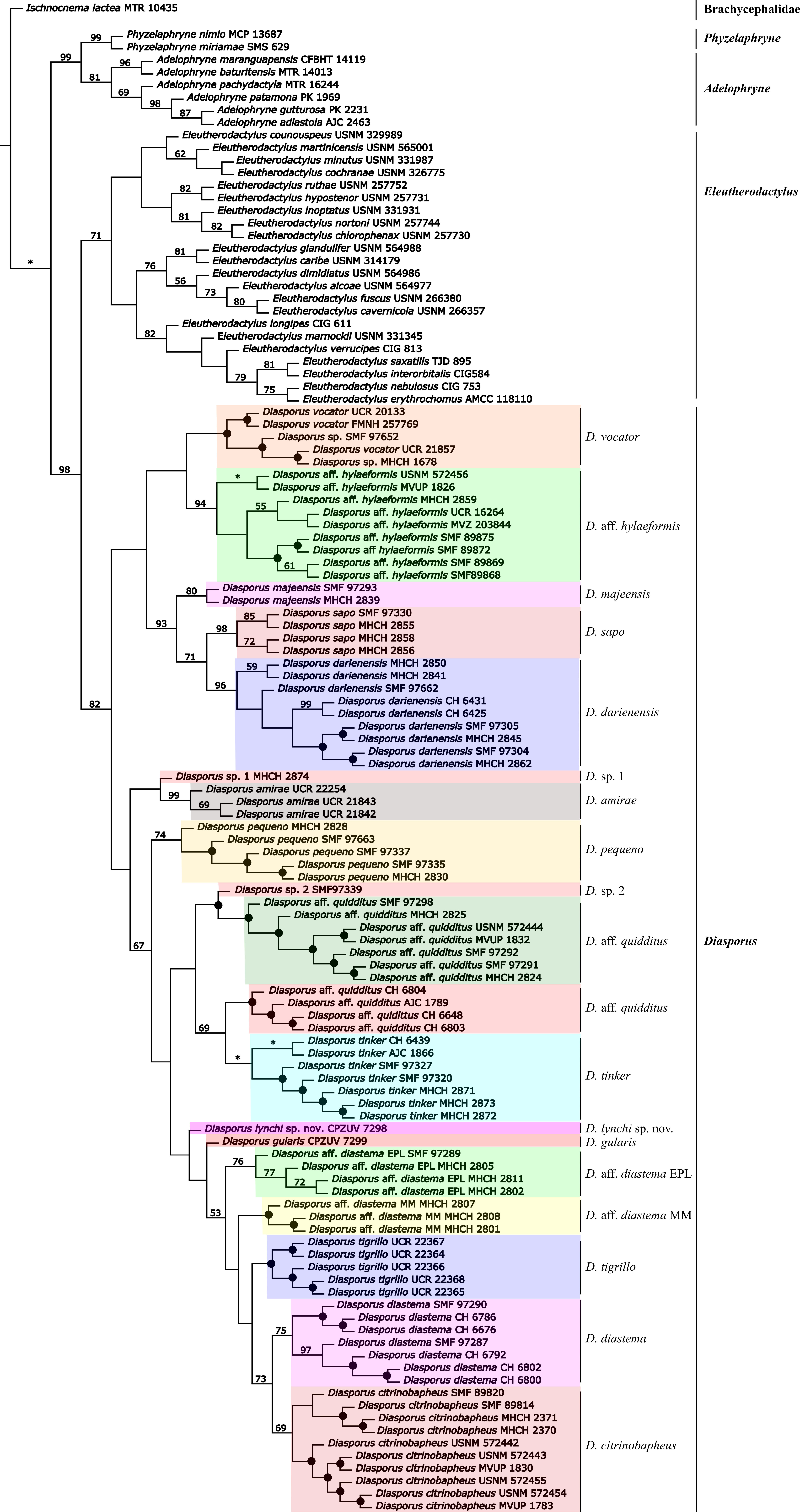

Supplement: Supplemental Information 5 — Black dots indicate nodes that collapse in the strict consensus. Numbers on nodes are Parsimony jackknife absolute frequencies values. Nodes without values indicate value <50% of jackknife value and an asterisk indicates a 100%. [file peerj-10-12765-s005.png]

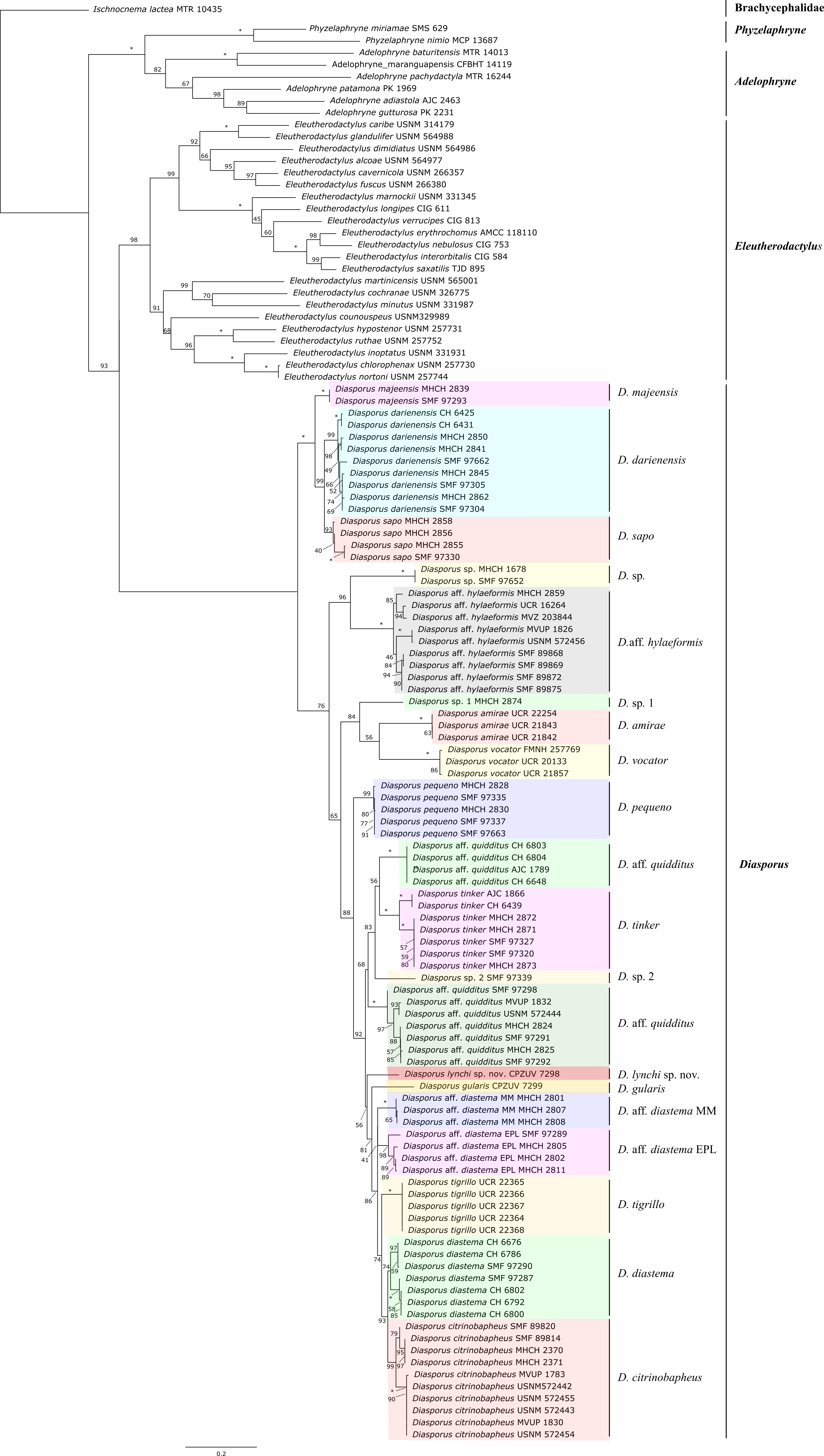

Supplement: Supplemental Information 6 — Numbers around nodes are the ultrafast bootstrap support values. An asterisk (*) indicates 100% ultrafast bootstrap support. [file peerj-10-12765-s006.png]
